# Transparent Bioplastic Derived from CO_2_-Based Polymer Functionalized with Oregano Waste Extract toward Active Food Packaging

**DOI:** 10.1021/acsami.0c12789

**Published:** 2020-09-21

**Authors:** Thi Nga Tran, Binh T. Mai, Chiara Setti, Athanassia Athanassiou

**Affiliations:** † Smart Materials, Istituto Italiano di Tecnologia, Via Morego, 30, Genova 16163, Italy; ‡ Istituto Italiano di Tecnologia, Via Morego, 30, Genova 16163, Italy

**Keywords:** poly(propylene carbonate), cellulose acetate, active packaging, antioxidant oregano, biodegradable plastic

## Abstract

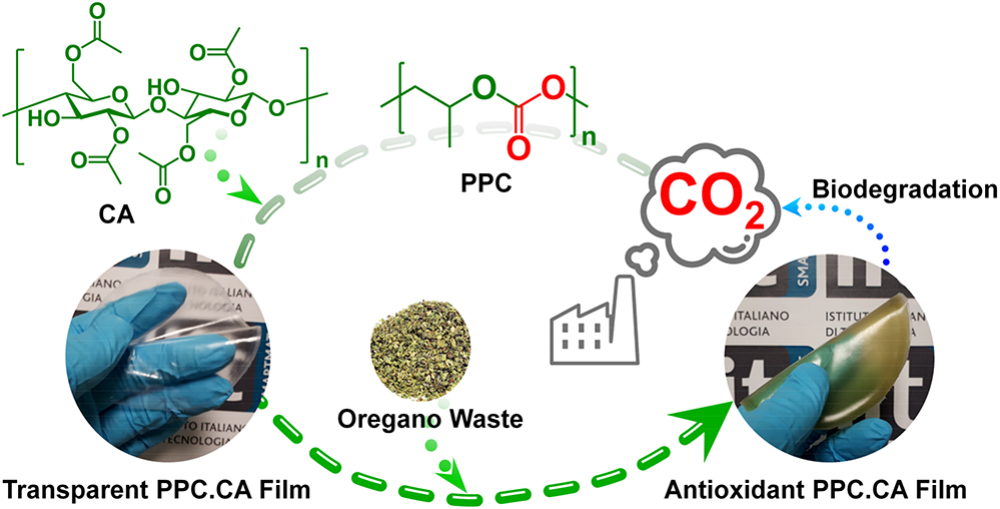

Active packaging materials, biodegradable and from renewable resources, are the most promising substitutes of nonbiodegradable, petroleum-based plastics, toward green and sustainable packaging solutions. In this study, an innovative bioplastic system, composed of carbon dioxide-derived poly(propylene carbonate) (PPC) and nature-originated cellulose acetate (CA), was developed. The extract from oregano waste was incorporated into the bioplastics as a low-cost and effective antioxidant resource. Thin, freestanding, and flexible PPC.CA bioplastic films were obtained by a simple, easily scalable solvent casting technique. The pristine films, without the oregano extract, featured good transparency and high water vapor barrier ability, along with suitable mechanical and thermal properties that are comparable to commercial plastics used for packaging. Interestingly, the incorporation of oregano waste extract added to the bioplastics high UV protection and high antioxidant activity, suitable features for active food packaging applications, without compromising the intriguing properties of the pristine films. The biocomposite films were not only biocompatible but also started biodegrading after just 1 week in seawater. The reported biocomposites are foreseen as promising candidates for several packaging applications, but in particular for sustainable active food packaging.

## 1 Introduction

Presently, worldwide plastic production has continuously grown, reaching 140 million tons annually for packaging applications alone.^1^ Most of the commercial packaging plastics, such as polyethylene terephthalate, polyethylene, polypropylene, polystyrene, etc., are synthetic, petroleum resource-based, non-biodegradable and non-biocompostable.^2−4^ The huge amount of waste generated after their short lifetime causes serious environmental issues, mainly due to their non-biodegradable nature. The most innovative and sustainable replacements are polymers from renewable resources, especially the ones that are also biodegradable, since their end of life biodegradability is combined with biocompatibility, the possibility of prior recycling, and environmental inertness.^5−8^ Among those, poly(propylene carbonate) (PPC) stands out as a very promising candidate, since it utilizes CO_2_ avoiding its disposal.^4,9^ PPC, an amorphous, thermoplastic, and aliphatic polycarbonate, is synthesized through the copolymerization of propylene oxide and CO_2_ gas.^10,11^ PPC features mechanical flexibility, good processability, transparency, excellent oxygen barrier properties, biodegradability, and biocompatibility, making it an interesting polymer for food packaging applications.^9,12,13^ Nevertheless, PPC has low thermal stability and relatively weak mechanical strength, properties that need to be improved prior to use of PPC as a practical packaging material.^4,14,15^ For this reason, numerous attempts have been focused on the development of biocomposites based on PPC. For instance, PPC has been combined with synthetic polymers and biopolymers like poly(lactic acid),^16^ poly(3-hydroxybutyrate-*co*-3-hydroxyvalerate),^17^ poly(*p*-vinylphenol),^18^ or with natural polymers including gelatin,^19^ chitosan,^12,20^ pristine, and thermoplastic starch.^9,21,22^ The thermal and mechanical performances of the obtained polymeric biocomposites were generally enhanced with respect to the individual polymers. However, in most cases, PPC was immiscible with other polymers, leading to phase separation, loss of transparency, and non-uniform films,^9,23^ thus hindering its applications in the packaging industry. Therefore, for the purpose of sustainable packaging applications, the development of biocomposite films based on PPC showing good transparency and sufficient thermal and mechanical performance is an emerging and active research area.

Cellulose acetate (CA), a common low-cost derivative of cellulose obtained by industrial scale esterification with glacial acetic acid and acetic anhydride in the presence of sulfuric acid,^24^ is an environmental friendly polymer that is promising for the packaging industry. In fact, CA has been widely exploited in several commercial products, including textiles, surface coatings, inks, eyeglass frames, cigarette filters, membranes, and packaging materials for baked goods and fresh products.^2,25,26^ CA features high heat resistance, great tensile strength, and rigidity, along with good dimensional stability.^27,28^ However, its high rigidity causes difficulties in thin film fabrication for packaging purposes. Thus, CA is generally compounded with plasticizers to obtain thermoplastic material with improved flexibility. However, the use of small molecules as plasticizers can cause leaching of these molecules out of the plastics to the packed food, with potential risks to consumer health as well as possible alterations to the taste and texture of food. As an alternative strategy, the production of blended polymeric composites composed of CA and other flexible biopolymers offers various advantages such as improved processability, enhanced mechanical properties, higher water vapor barrier, and not releasing harmful compounds.^7,27^ Therefore, the biocomposites of PPC and CA can be expected to hybridize their useful characteristics, resulting in intriguing sustainable polymeric packaging materials.

In comparison to conventional packaging materials, the active ones show desirable benefits, mostly their ability to maintain freshness and prolong food shelf life.^29,30^ Presently, due to various factors including marketing purpose, consumer demand, and the potential health issues and toxicological side effects of artificial active additives, there is an increasing interest in the use of safe and natural antioxidant materials as additives for active packaging.^29,31,32^ Oregano (*Origanum vulgare*) has been used as a common herb for centuries in daily cuisine.^33^ Oregano provides numerous health benefits thanks to its abundant amount of antioxidants, namely, rosmarinic acid, carvacrol, thymol, limonene, quercetin, pinene, ocimene, caryophyllene, and other polyphenols, flavones, and flavonols.^5,33^ Furthermore, its extract can be incorporated into polymeric films to obtain active packaging materials. Indeed, the incorporation of oregano extract into synthetic nonbiodegradable polymeric films, according to an innovative patented procedure,^34^ resulted in packaging films that showed significant improvement of the oxidative stability of the packed lamb steaks by extending their fresh odor and color from 8 to 13 days compared to the control.^30^ Besides that, active oregano-based films also showed significant increase of the packed fresh beef shelf life from 14 to 23 days.^35^

Generally, commercialized oregano contains its dried leaves and flowers.^6^ The branches of oregano are usually discarded as a byproduct of dried oregano production, but they still contain numerous valuable antioxidant compounds to be used as a low-cost natural antioxidant, integrated in bioplastics for active packaging application. In the race to discover sustainable polymeric composites toward active packaging, in this study, we introduce transparent and flexible biocomposite films based on biodegradable CO_2_-derived PPC and CA with the incorporation of oregano waste extract from discarded oregano branches. The morphology; light transmittance; chemical, mechanical, and thermal properties; and water vapor permeability, hydrophobicity, biodegradability, and antioxidant capacity of the developed films have been profoundly characterized to validate their potentiality as active food packaging materials.

## 2 Results and Discussion

After dissolving both PPC and CA in a common relatively benign solvent, their homogeneous mixtures were cast in molds, leaving the solvent to evaporate in controlled ambient conditions. The obtained thin bioplastic films were named as PPC.CA *x*. *y*, where *x* and *y* are the weight percentages of PPC and CA inside the biocomposite films, respectively. The films incorporating oregano waste extract (ORE) were prepared in a similar way and contained 7.98 wt % of oregano waste extract with respect to the polymers.

### 2.1 Chemical Interactions Characterization

**Figure 1:**
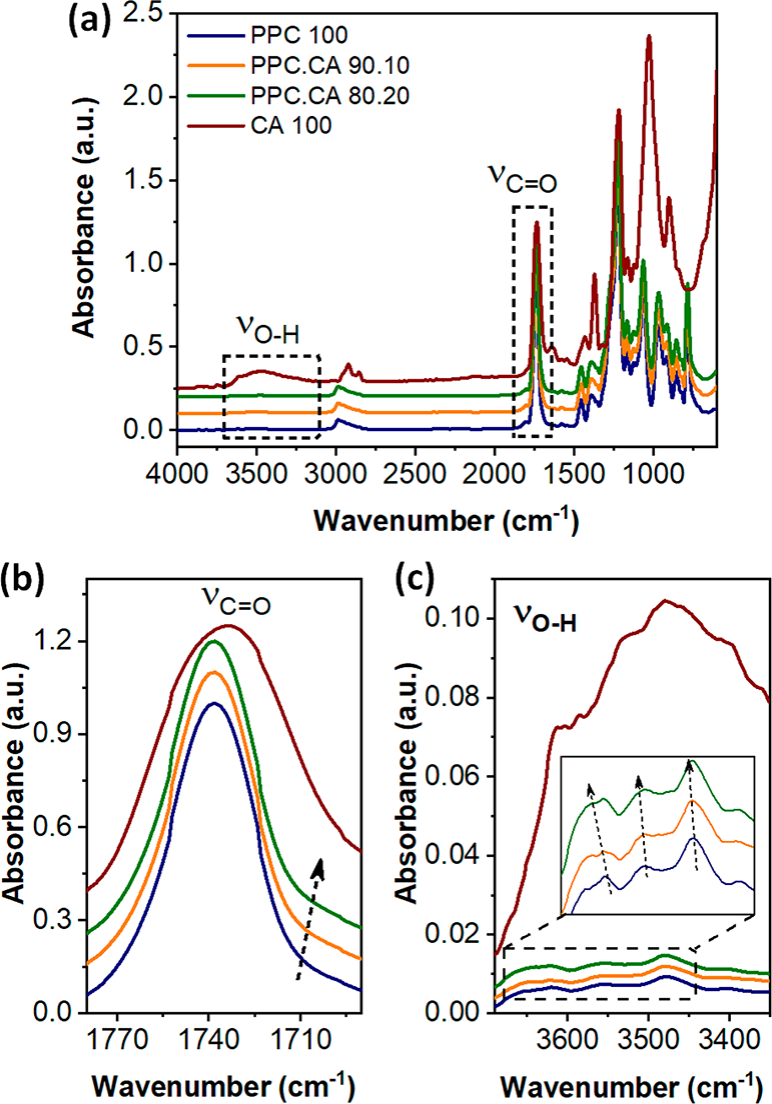
(a) FTIR spectra of pure PPC, pure CA, and PPC.CA biocomposites films. The detailed FTIR spectra of biocomposite films in (b) the carbonyl stretching region and (c) the hydroxyl stretching region. The black arrows in the in insets of (b,c) indicate the shifting of these peaks.

Generally, when two polymers are compatible or partly miscible, specific chemical interactions are formed between the chains of the two polymers in the entire polymers’ backbone or at the interfaces between the two polymer phases.^7,18^ These new interactions lead to changes in the FTIR spectra of the composite (i.e., band shifts, broadening peaks) in comparison to the FTIR spectra of the individual polymers.^7,18^ In order to investigate the possible chemical interactions between PPC and CA polymers, the FTIR spectra of the PPC.CA bioplastic films without oregano waste extract are shown in Figure 1a. The characterization was focused on two main regions of hydroxyl (ν_O–H_) and carbonyl (ν_C=O_) stretching vibrations since they are the most sensitive regions for hydrogen bonding interaction in the FTIR spectra.^7,32^ For direct comparison, all the spectra were normalized to the carbonyl stretching peak. Figure 1b shows the spectra of PPC, CA, and PPC.CA bioplastics in the carbonyl stretching region. The C=O stretching band of PPC, PPC.CA 90.10, and PPC.CA 80.20 is visualized as a strong and sharp peak centered at 1736 cm^–1^, while the one of CA is located at 1732 cm^–1^. As observed in the inset of Figure 1b, increasing the CA percentage in the bioplastics, the carbonyl stretching peaks of the PPC.CA films are broadened toward lower wavenumbers resulting in a small shoulder around 1703 cm^–1^ (indicated by the arrow). These changes in the carbonyl stretching region of PPC are due to the formation of new hydrogen bonded C=O. For an easier observation of such small peak shifts, the FTIR spectra in the regions of C=O and O–H stretching bands are presented superimposed in Figure S1a,b (Supporting Information, SI). As seen also from Figure S1a, the broadening of the carbonyl stretching peak is observed exclusively toward low wavenumbers. This observation confirms that the shift in the C=O peak of the composites is not due to the convolution of C=O bands of PPC and CA, but it is due to the formation of new hydrogen bonds between the individual polymers.

In the 3700–3100 cm^–1^ hydroxyl stretching region in Figure 1c, the absorption of the bioplastic films containing PPC is negligible, due to the low number of terminal O–H groups in the PPC chain ends.^15^ However, thanks to the abundant hydroxyl groups on CA polymer structure, the FTIR spectrum of CA shows an intense hydroxyl stretching band centered at 3484 cm^–1^. The inset in Figure 1c shows that the absorption of the PPC O–H stretching vibration shifts to higher wavenumbers when the amount of CA in the films increases, as illustrated by the arrows.

**Figure 2:**
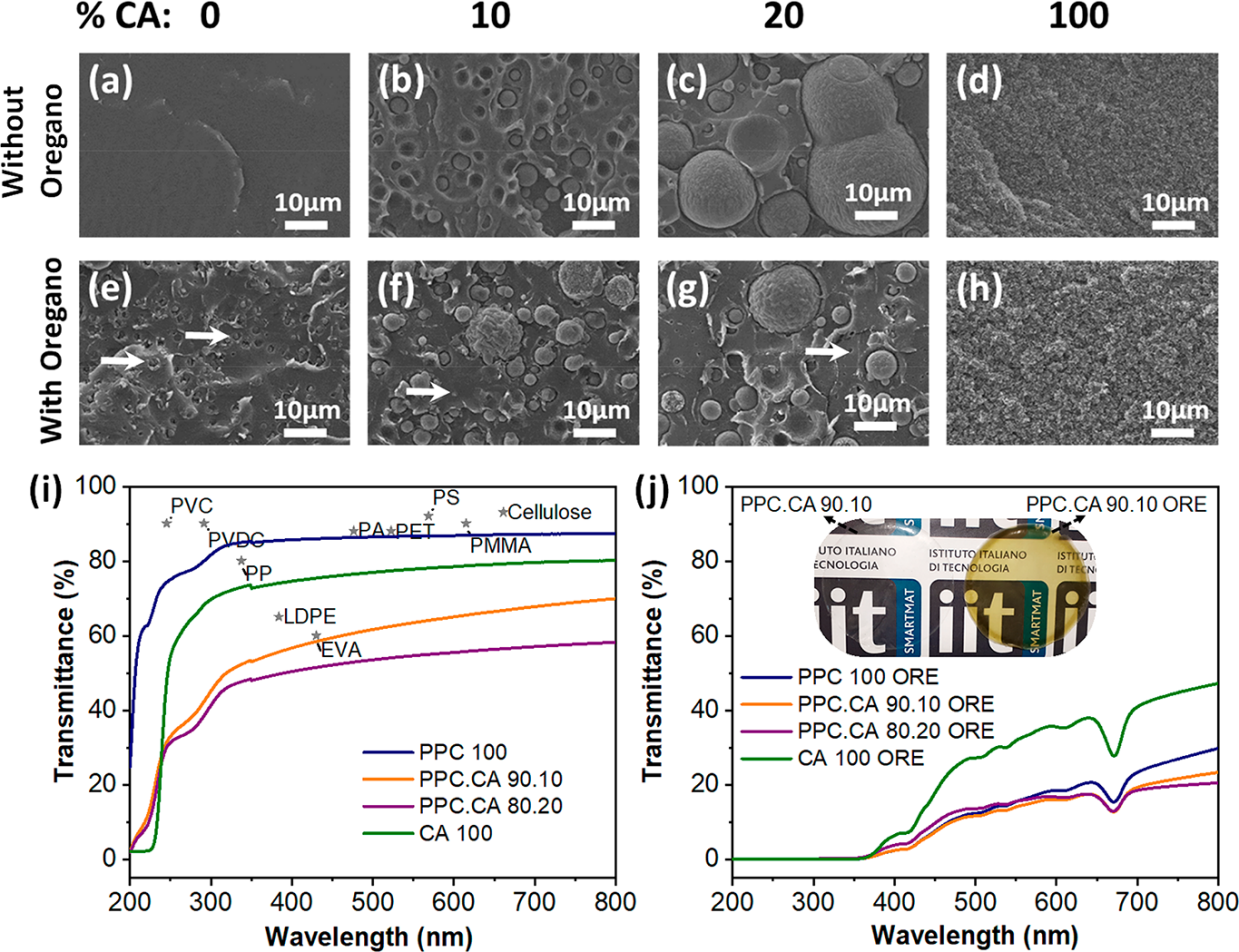
SEM images showing the morphology of the cross sections of cryo-fractured films of (a) pure PPC, (b) PPC.CA 90.10, (c) PPC.CA 80.20, and (d) pure CA, as well as (e–h) the respective films containing oregano waste extract. UV–vis spectra of (i) pure PPC 100, CA 100 and PPC.CA bioplastic films as well as (j) respective films containing oregano waste extract. For comparison, the maximum transmittance values of commonly used polymers^7,31,39^ in packaging applications are included in (i) (the positions on *x*-axis are random). Inset in (j) shows a photograph of two films composed of 90% PPC and 10% CA: the transparent colorless film does not contain oregano extract, while the transparent greenish film contains oregano extract. Abbreviations: PVC, polyvinyl chloride; PVDC, polyvinylidene chloride; PP, poly propylene; LDPE, low-density polyethylene; EVA, ethylene vinyl acetate; PA, polyamide; PET, poly(ethylene terephthalate); PS, poly stryrene; and PMMA, poly(methyl methacrylate).

To summarize, the FTIR analysis shows that the absorption of the hydroxyl stretching vibration shifts to higher wavenumbers, whereas the carbonyl peaks are broadened toward lower wavenumbers. Hence, intermolecular hydrogen bonding interactions are formed between the O–H end groups of PPC and C=O of CA polymer chains, even though the changes they induce to the FTIR spectra are modest due to the low amount of the new H-bonds. These limited and weak hydrogen bonds could be established on the interfaces of PPC matrix and CA microparticles (see the micromorphologies next, in Figure 2, SEM). Similar newly formed weak interactions have been reported for blends of PPC and bisphenol A or poly(*p*-vinylphenol).^15,18^ Thus, PPC and CA are quite incompatible polymers resulting in slight changes in their vibration peaks. The shifting of O–H and C=O stretching bands was also found in the biocomposite films containing oregano waste extract (Figure S2), and it was more intense with respect to the composite films without oregano (Figure S2d), indicating the formation of extra hydrogen bonding between the O–H end groups of PPC and the C=O groups of the abundant small molecule antioxidants in oregano waste extract such as phenolic acids and flavonoids.^15,36,37^

### 2.2 Micromorphology of CO_2_-Based Bioplastic Films

In order to investigate the microstructure of the PPC.CA bioplastic films, their surface and cross-section morphologies were characterized by SEM. All PPC.CA bioplastic films with or without oregano waste extract showed flat, smooth, and homogeneous surfaces (Figure S3). The microstructures of cryo-fractured cross sections of the bioplastic films are displayed in Figure 2a–h. Pure PPC films (Figure 2a) featured a continuous, densely packed and smooth structure throughout their volume. While, as seen in Figure 2d, pure CA films showed cross sections with homogeneous microporous structure. In the composite films, CA was incorporated into the PPC, and thus, CA microparticles were formed in the compact PPC matrix (Figure 2b,c). The size of CA particles increased with the percentage of CA in the films ranging from around 4 to 20 μm, as demonstrated in Figure 2b,c for PPC.CA 90.10 and 80.20, respectively. This phenomenon is commonly reported for PPC-based polymeric composites.^9,18,23^ This observation is in agreement with the FTIR analysis (Figure 1) where limited hydrogen bonding interactions were found between PPC and CA polymer chains, indicating partial miscibility of the two polymers at their interfaces.

In the case of the bioplastic films containing oregano waste extract (Figure 2e–h), the presence of small particles of around 1.5 μm (indicated with white arrows) from dried oregano powder, which passed through the filter paper during the filtration of oregano extract, was observed in all the samples. The extract incorporation seems to affect the structure of the individual polymers, since small particles from the extract partly interrupt the polymer matrices’ continuity (Figure 2, parts a vs e and d vs h), whereas it does not significantly modify the morphology of the composite bioplastic PPC.CA film (Figure 2, parts b vs f and c vs g), where the PPC matrix appeared already interrupted by the CA microparticles. XDR measurements were also performed on the PPC.CA bioplastics with and without oregano waste extract. It turned out that the biocomposites are amorphous with their XRD patterns being the superposition of the PPC and CA patterns (see SI Figure S4). In the case of the biocomposites with oregano extract a small crystalline peak, around 13°, is due to the crystalline cellulose in oregano microparticles.^7,38^

### 2.3 Optical Properties

The consumer acceptance of a product largely depends on the appearance of the packaging material, in particular color, transparency, and gloss. However, the optical characteristics of the packaging materials can also affect their protection ability toward food, since the exposure of the food to visible and ultraviolet (UV) light can lead to loss of nutrients and flavor. Thus, optical properties are among the most important properties of food packaging materials. The transmission of both UV and visible light through the PPC.CA bioplastic films in the wavelength range 200 to 800 nm is shown in Figure 2i,j. In the range of visible light (350–800 nm), pure PPC and CA films presented slightly higher transmittance in comparison to the PPC.CA films (Figure 2i). In detail, the biocomposite films PPC.CA 90.10 and 80.20 showed 67.9 and 57.1% transmittance at 700 nm, respectively, whereas for PPC and CA the respective values were 87.3% and 79.7%. Such properties can be explained upon observation of the microstructure of the hybrid films in the SEM images in Figure 2a–h. The presence of CA particles modified the interaction between the light and the bioplastic PPC.CA films, since the CA microparticles act as light scattering centers, affecting the film transparency. Higher CA percentage resulted in denser packing of the CA particles as well as higher variability of the particles’ size (Figure 2b,c) increasing the centers of obstruction of light transmission. Furthermore, all the pristine CO_2_-based bioplastic films showed comparable transparency with respect to commercial polymers (Figure 2i) which are commonly used in food packaging applications, such as polyvinyl chloride (PVC), poly propylene (PP), low-density polyethylene (LDPE), poly(ethylene terephthalate) (PET), poly styrene (PS), etc.^7,31,39^ This result suggests that the developed PPC.CA bioplastic films can be employed as transparent food packaging materials. The physical appearance of a PPC.CA 90.10 film is displayed in the inset of Figure 2j, illustrating its excellent transparency.

When the oregano waste extract was introduced into the bioplastic films, the resulting films acquired a light green color, as shown in the inset of Figure 2j for PPC.CA 90.10 ORE. It can be seen from the UV–vis spectra in Figure 2j that in comparison to the pristine films without oregano extract (Figure 2i), the bioplastic films incorporating oregano waste extract, still being transparent, show lower transmittance in the visible light range from 350 to 800 nm, providing higher light protection to the packaged food. The increase of opacity is generally observed in several films containing plant-based extracts such as olive waste or ginger, rosemary, black tea, and green tea extracts.^5,40^ More importantly, as seen in Figure 2j, the CO_2_-based bioplastic films incorporating oregano waste extract can act as excellent UV barrier, since in the UV range of 200–350 nm, they showed negligible UV light transmission. This can be attributed to the high content of aromatic compounds in the oregano waste extract (for instance, caffeic acid, rosmarinic acid, carvacrol, and other polyphenols) which absorb UV light, acting as excellent UV barrier materials. This characteristic can help in the prevention of UV light-induced lipid oxidation that occurs during the exposure of the packed food products to UV light. Hence, the presence of oregano waste extract in the bioplastic films enhances the UV light protection ability of the packaging films, at the same time retaining the possibility to observe the packaged food through them, and thus it is expected to contribute significantly to the food shelf life extension preserving the consumer-requested transparency of the packaging.

### 2.4 Mechanical Properties

**Figure 3:**
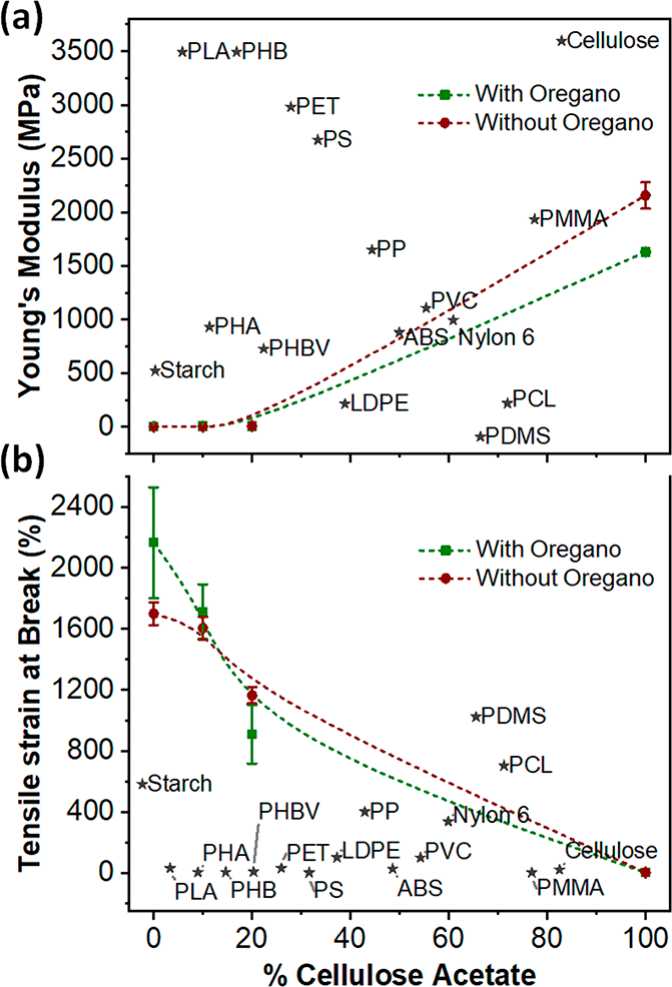
Mechanical properties ((a) Young’s modulus and (b) Tensile strain at break) of pure PPC, pure CA, and PPC.CA bioplastic films as well as films containing oregano extract. The dashed lines are to guide the eye. For comparison, the Young’s modulus and tensile strain at break of commonly used polymers for packaging application are included^7,8,31,32^ (the position of the various polymers with respect to the *x*-axis is random). Abbreviations: Starch, thermoplastic starch; PLA, poly(l-lactic acid); PHA, polyhydroxyalkanoates; PHB, poly(hydroxybutyrate); PHBV, poly(hydroxybutyrate-*co*-hydroxyvalerate); PET, poly(ethylene terephthalate); PS, poly stryrene; LDPE, low-density polyethylene; PP, poly propylene; ABS, acrylonitrile butadiene styrene; PVC, polyvinyl chloride; PDMS, polydimethylsiloxane; PCL, poly(caprolactone); and PMMA, poly(methyl methacrylate).

For food packaging, the mechanical behavior of the packaging films is a crucial factor since it requires both sufficient resistance and flexibility to facilitate handling and avoid damage during packaging, transport, and storage periods. Full tensile strain–stress curves of pure PPC, pure CA, and PPC.CA bioplastic films as well as films containing oregano extract are presented in SI Figure S5. The Young’s modulus and tensile strain at break of PPC.CA bioplastic films with and without oregano extract are presented in Figure 3a,b, respectively. Pure PPC 100 films showed a low Young’s modulus (1.82 ± 0.27 MPa), demonstrating their ductile character. Due to the highly rigid nature,^7^ CA 100 displayed a quite high Young’s modulus value (2155.27 ± 123.96 MPa), which is comparable to commercialized PP, PMMA, and PVC plastics,^7,8,31,32^ as depicted in Figure 3a. Surprisingly, despite the formation of CA microparticles (SEM images, Figure 2) and the partial miscibility of the two biopolymers, the incorporation of CA into PPC matrix helped to improve the stiffness of the resulting films. The well-dispersed CA microparticles apparently play the role of effective fillers and cross-linking centers via weak hydrogen bonding (proved by FTIR, Figure 1), resulting in the toughening effect. In particular, in comparison to pure PPC film (1.82 ± 0.27 MPa), the Young’s modulus of bioplastic films containing 10 and 20 wt % of CA increased to 2.37 ± 0.26 MPa and 6.86 ± 1.93 MPa, indicating 30% and 277% enhancement, respectively. The addition of oregano extract did not significantly modify the micromorphology and crystalline patterns (SEM (Figure 2) and XRD (Figure S4)) of the obtained bioplastic films, thus, as expected, no considerable differences in the Young’s modulus of PPC.CA ORE films were observed in comparison to PPC.CA films. This result is consistent with the SEM analysis above (Figure 2a–h), which showed practically identical structures between bioplastic films with and without oregano waste extract.

In addition, the theoretical predictions of Young’s moduli of PPC.CA and PPC.CA ORE films were calculated using formulas 3 and 4 based on the rule of mixtures (see Experimental Section).^41,42^ The calculated highest upper bound (*E*_U_) and lowest lower bound (*E*_L_) and the corresponding measured Young’s modulus (*E*_M_) are listed in Table 1 for comparison. As shown, the *E*_U_ values from the so-called parallel model are far higher than the experimental *E*_M_. However, the lowest lower bounds *E*_L_ are somewhat closer to the Young’s modulus. Particularly, for PPC.CA 90.10, the measured Young’s modulus is very close to the theoretical *E*_L_, whereas for PPC.CA 80.20 the measured Young’s modulus is almost three times higher than *E*_L_. In general, the series model is more precise to predict the Young’s modulus of the polymeric composites containing micro- and/or nanosized reinforced fillers.^41,43^ Here, as observed in the SEM images, the CA microparticles are well distributed within the PPC polymer matrix and thus are in good agreement with the theoretical calculations using the series model. Moreover, as depicted in Figure 3a, all the developed CO_2_-based bioplastics show comparable Young’s moduli with popular polymers employed for food packaging applications.

**Table 1:** Experimental Young’s Modulus (*E*_M_) and Theoretical Predictions (Highest Upper Bound *E*_U_ and Lowest Lower Bound *E*_L_) of PPC.CA Biocomposite Films with and without Oregano Waste Extract

	Young’s modulus of films without oregano (MPa)			Young’s modulus of films with oregano (MPa)		
CA percentage (%)	*E*_M_	*E*_U_	*E*_L_	*E*_M_	*E*_U_	*E*_L_
10	2.37	211.18	2.02	4.69	159.65	1.57
20	6.86	421.84	2.26	5.68	318.86	1.76

a The highest upper bound *E*_U_ and the lowest lower bound *E*_L_ were calculated from formulas 3 and 4 (Experimental Section).

Figure 3b displays the strain at break of all PPC.CA bioplastic films. Interestingly, the pure PPC bioplastic films showed excellent stretchability with a tensile strain at break of 1700 ± 74%, which is almost double that of PDMS. Pure CA films showed, on the contrary, quite low elongation of 4.9% demonstrating high brittleness and fragility. The elongation ability of PPC.CA decreased with the increase of CA percentage. The results can be explained by the formation of bigger CA particles (Figure 2, SEM) which somehow interrupt the continuous PPC matrix, hence, the obtained films had lower elongation ability. Nevertheless, the tensile strain at break of PPC.CA 80.20 films was still considerably high at 1165 ± 54%, which is greater than the elongation of other common plastics^7,8,31,32^ used in food packaging (Figure 3b). In line with the above SEM and Young’s modulus data, the introduction of oregano extract did not significantly alter the elongation ability of PPC.CA bioplastic films, as can be observed in Figure 3b. Thus, the mechanical properties of the developed PPC.CA bioplastic films are sufficient for food packaging application and comparable with other commercial food packaging plastics. In particular, the developed CO_2_-based bioplastics showed exceptionally high elongation at break, which is advantageous for flexible packaging.

### 2.5 Thermal Stability

**Figure 4:**
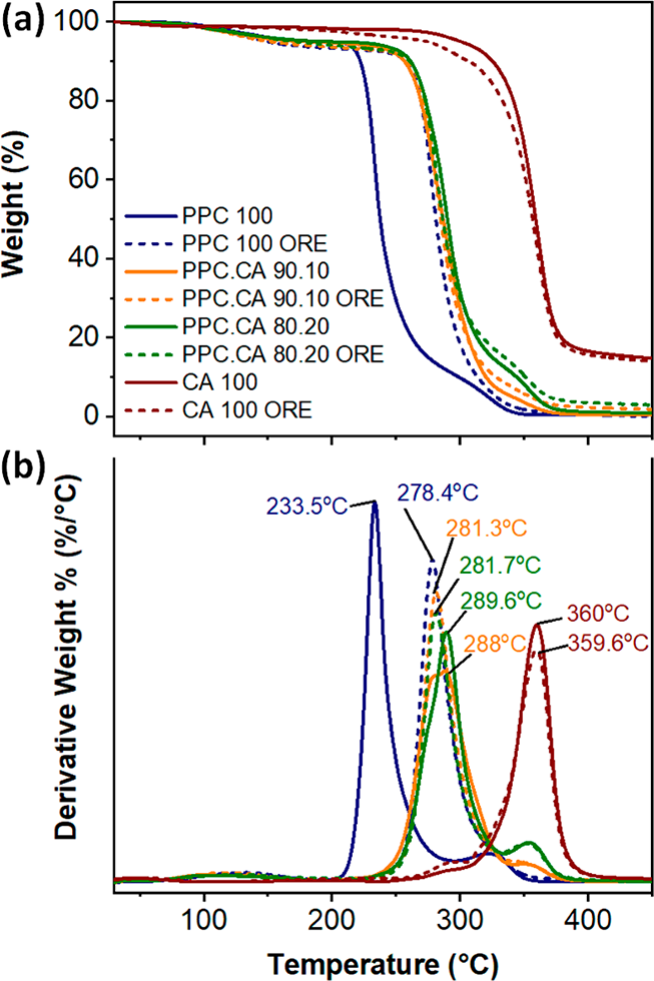
(a) TGA thermal weight loss as a function of temperature plots of pure PPC, pure CA, and PPC.CA bioplastic films (solid lines), as well as of the respective films containing oregano waste extract (dashed lines). (b) Derivative of the thermograms presented in (a) with detailed degradation temperatures.

The thermal stability of CO_2_-derived bioplastic films was characterized by TGA measurements. The thermal diagrams and their corresponding derivative plots of pure PPC, pure CA, and PPC.CA bioplastic films along with the respective films containing oregano waste extract are displayed in Figure 4a,b, respectively. All the CO_2_-based bioplastic films exhibited high resistance to thermal degradation with decomposition temperatures higher than 200 °C. In detail, pure PPC films showed the lowest thermal degradation temperature at 233.5 °C. Meanwhile, pure CA films thermally degraded at 360 °C, demonstrating the highest stability among all the bioplastic films. The presence of CA increased the thermal stability of the resulting biocomposite films. The thermal degradation points of PPC.CA films 90.10 and 80.20 shifted to 288 and 289.6 °C, respectively, indicating an increase of about 55 °C in comparison to pure PPC films. In addition, it is worth mentioning that the thermal degradation temperature of PPC films loaded with oregano waste extract also increased to 278.4 °C, which is 45 °C higher compared to PPC films. It was reported that PPC is thermally decomposed to cyclic propylene carbonate by unzipping mechanism, in which, the free hydroxyl end group reacts with nearby carbonate group to form stable 5-member ring compound called 4-methyl-1,3-dioxolan-2-one.^23,44,45^ Thanks to the hydrogen bonding interactions formed between the hydroxyl end-groups of PPC and the carbonyl groups of CA polymer chains, as discussed in FTIR analysis (Figure 1), as well as the steric hindrance caused by the presence of CA within the PPC matrix, the decomposition (carbonate biting) reaction delayed and required higher temperatures.^45^ The enhanced resistance to thermal degradation of PPC 100 ORE can also be deduced by the presence of hydrogen bonding interactions between the hydroxyl end-groups of PPC and the carbonyl groups of the abundant small antioxidant molecules in oregano waste extract^15,36,37^ as discussed in the FTIR analysis (Figure 1 and Figure S2). Hence, the thermal stability of PPC polymer was improved in PPC.CA and PPC.CA ORE biocomposites. To this point, the hydrogen bonding between the −OH groups at PPC chain ends and the −C=O of CA can be confirmed from the results of both FTIR and TGA.

### 2.6 Water Vapor Permeability

**Figure 5:**
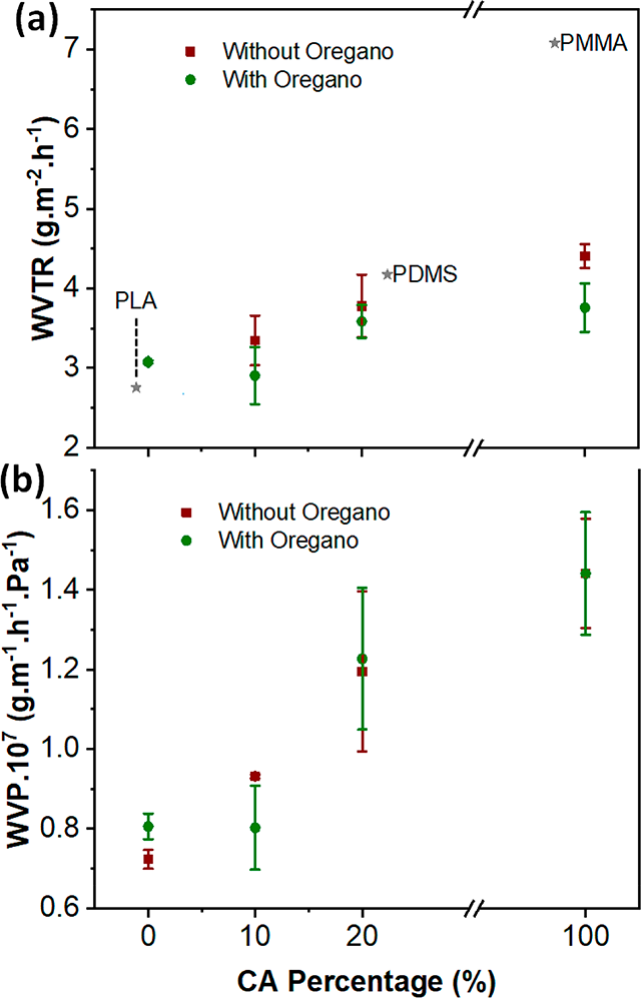
(a) Water vapor transmission rate (WVTR) and (b) water vapor permeability (WVP) as a function of CA percentage of pure PPC, pure CA, and PPC.CA bioplastic films as well as films containing oregano waste extract. The WVTR values of common polymers are included in (a) for comparison; the positions on *x* axis are random. Abbreviations: PLA, poly(l-lactic acid); PDMS, polydimethylsiloxane; and PMMA, poly(methyl methacrylate).

The permeation of water vapor through the developed bioplastic films, an important factor of packaging materials, was investigated. The results of water vapor transmission rate (WVTR) and water vapor permeability (WVP) as a function of CA percentage in the films are presented in Figure 5a,b, respectively. As shown in Figure 5a for the bioplastics without oregano waste extract, pure PPC films showed the lowest WVTR of 2.702 ± 0.920 g·m^–2^·h^–1^ thanks to the hydrophobic nature and dense structure of this polymer (SEM, Figure 2a), while the WVTR of CA was the highest with a value of 4.409 ± 0.148 g·m^–2^·h^–1^. The WVTR increased with the increasing amount of CA in the PPC bioplastic, to 3.349 ± 0.312 g·m^–2^·h^–1^ and 3.702 ± 0.994 g·gm^–2^·h^–1^ for PPC.CA 90.10 and 80.20, respectively. The incorporation of oregano waste extract did not remarkably alter the WVTR of the resulting films due to the almost unaltered structure of the films (SEM, Figure 2). Interestingly, as depicted in Figure 5a, the PPC.CA bioplastic films exhibited comparable WVTR to poly(lactic acid), which is one of the most widely used biodegradable polymers for food and other packaging applications^31,46^ and also to polydimethylsiloxane film, which is broadly used in sealing applications.^7,31,32^

The water vapor permeability (WVP) in Figure 5b also showed an increasing trend with increasing percentage of CA in the films. No significant difference in WVP was observed for the films with and without oregano waste extract. In addition, the behavior of the developed biocomposite films when in contact with water droplets were also characterized, and the results are presented in SI Figure S6.

### 2.7 Antioxidant Activity

**Figure 6:**
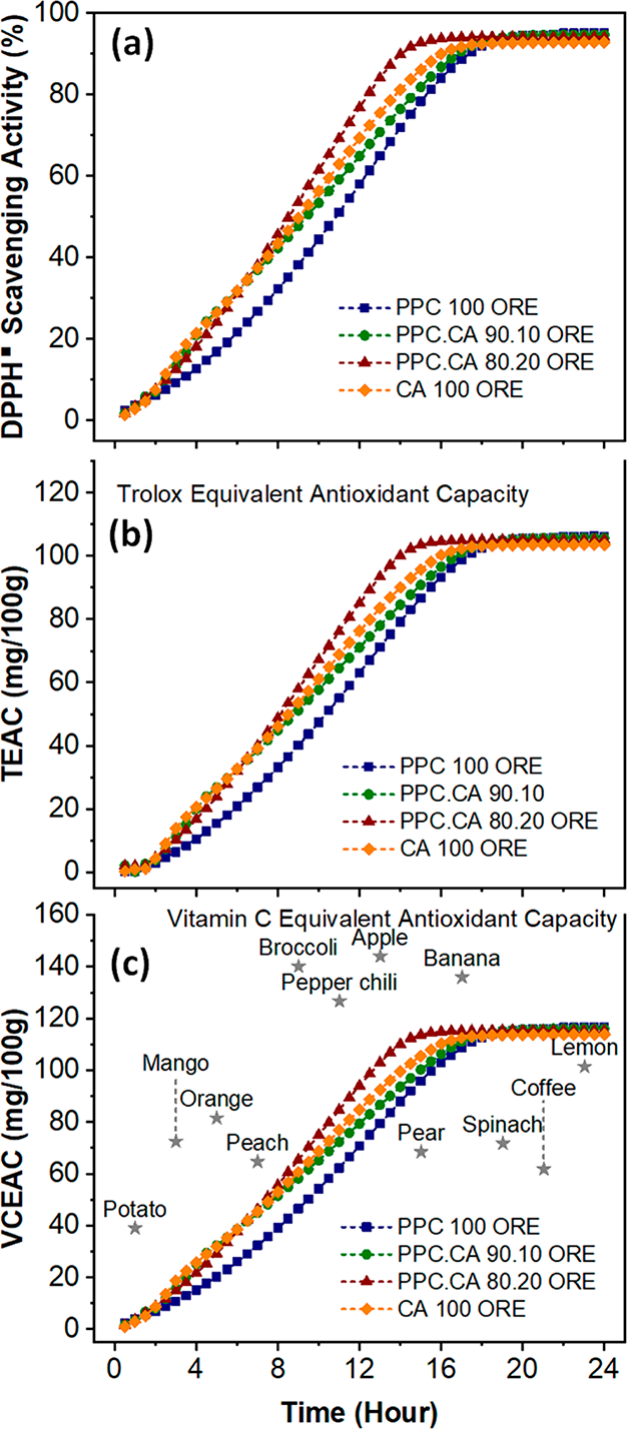
(a) DPPH^•^ free radical scavenging activity, (b) Trolox (TEAC) and (c) Vitamin C equivalent antioxidant capacity (VCEAC) of PPC.CA bioplastic films containing oregano waste extract. The VCEAC of popular antioxidant-rich foods are included in (c) for comparison (the position of the various polymers with respect to the *x*-axis is random).

To turn standard packaging into anactive one, the antioxidant performance of the material is a crucial factor. It is well-known that oregano contains numerous valuable antioxidants, including phenolic acids such as caffeic, coumaric, ferulic, and neochlorogenic; along with flavonoids like quercetin, luteolin, apigenin, kaempferol, and isorhamnetin.^36,37^ Such compounds, having unique chemical structures, exhibit several interesting biological activities, such as radical scavenging capability, antimicrobial, antibacterial, and antiseptic properties.^36,47^ In this study, the scavenging activity against DPPH^•^ free radical was used to determine the antioxidant capacity of the developed bioplastic films, as shown in Figure 6a. The antioxidant activity of the films was also compared with artificial (Trolox) and natural (Vitamin C) antioxidants^48^ in Figure 6b,c, respectively. The calibration curves of DPPH• scavenging activities of Trolox and Vitamin C at various concentrations are shown in SI Figure S7. Figure 6a presents the DPPH^•^ free radical scavenging activity as a function of time of all CO_2_-based biocomposite films containing oregano extract. All the films showed a continuous and gradually increased antioxidant activity with time. The PPC.CA ORE films with higher CA content exhibited faster scavenging ability. Particularly, the times to reach 50% DPPH^•^ scavenging activity for PPC 100 ORE, PPC.CA 90.10 ORE, PPC.CA 80.20 ORE, and CA 100 ORE were 11.0, 9.0, 8.5, and 9.5 h, respectively. This could be attributed to the presence of more and larger CA microparticles within the PPC matrix (SEM, Figure 2a–h) of the composite films containing higher CA percentage, which help to diffuse and release the antioxidant compounds more efficiently since CA has higher affinity to water compared to PPC. Furthermore, the maximum antioxidant activity of the bioplastics was equivalent to 105 mg Trolox/100 g film, and 115 mg Vitamin C/100g film, as can be observed from Figure 6b,c. Figure 6c shows that the Vitamin C equivalent antioxidant capacity (VCEAC) of PPC.CA ORE bioplastic films was comparable to those of well-known antioxidant-rich foods, and even better in some cases, for instance with respect to orange, lemon, coffee, and spinach, etc.^48−50^ Due to their excellent antioxidant activity, the developed PPC.CA ORE biocomposite films could be employed as flexible and active food packaging materials for fresh meat, poultry, or fish, as reported for other active packaging film.^30,35,51^

### 2.8 Biodegradability in Seawater

**Figure 7:**
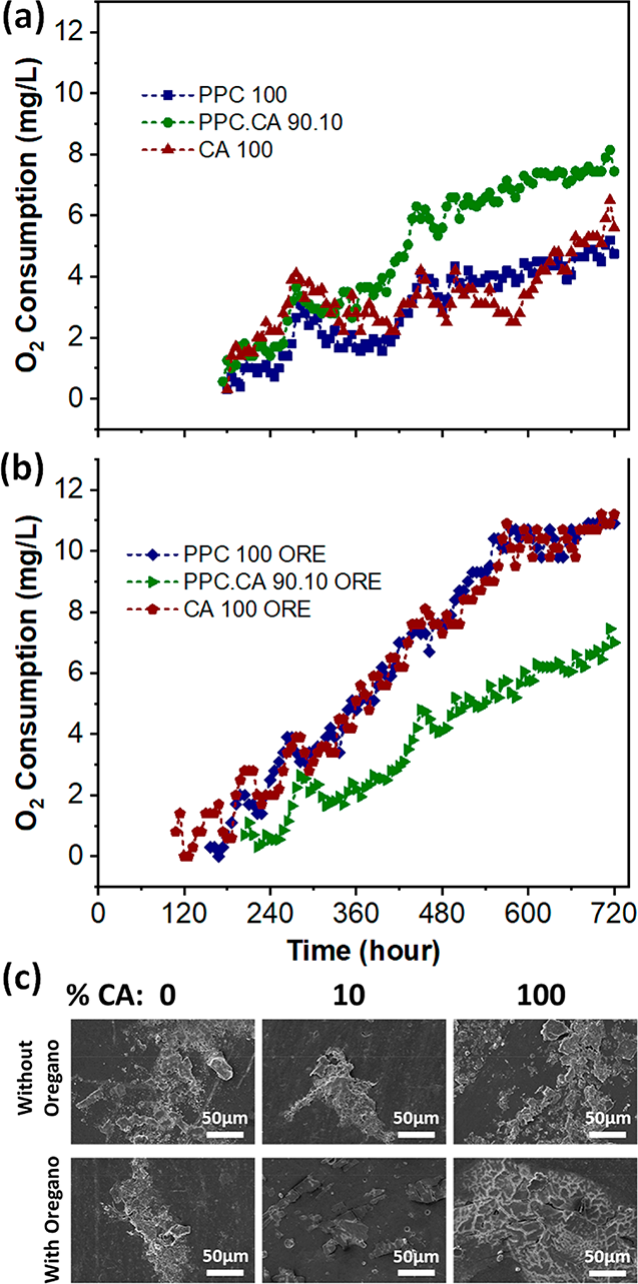
(a,b) Oxygen consumption during BOD experiments and (c) surface morphologies of pure PPC, pure CA, and PPC.CA 90.10 bioplastic films as well as films containing oregano extract after 30 days in seawater for the BOD test.

The ability of selected PPC.CA bioplastic films to biodegrade in seawater was evaluated by the measurement of oxygen consumption in the biodegradation reaction during 30 days of BOD experiments. Figure 7a,b shows the oxygen consumption amount as a function of time of bioplastic films with and without oregano extract, respectively. It is noteworthy that the biodegradation of PPC produces only benign and nontoxic compounds including water and CO_2_.^9,11,21,22^ The pure PPC, CA, and PPC.CA 90.10 films started to biodegrade after 180, 180, and 174 h (∼7.5 days), respectively, in seawater at ambient temperature (about 25 °C) and in the dark. The maximum oxygen consumption in the case of PPC, CA, and PPC.CA 90.10 reached 5.18, 6.45, and 8.12 mg/L, respectively. Thus, in comparison to pure PPC and CA films, PPC.CA 90.10 biocomposite films began to biodegrade in a shorter time with higher oxygen consumption, indicating an improvement of biodegradability. This feature was obtained thanks to the formation of CA microparticles in the structure of PPC.CA 90.10 films, which helps the seawater and microorganisms to penetrate inside the films, thus, facilitating the biodegradation process.

When oregano waste extract was incorporated in the films, the pure polymer ones started the biodegradation in seawater earlier compared to the composite PPC.CA 90.10 films showing also higher oxygen consumption over time. Indeed, the biodegradation in seawater began for PPC 100 ORE, CA 100 ORE, and PPC.CA 90.10 ORE after 156, 108, and 204 h, respectively. This behavior is most likely connected to the change in the structure of the pure PPC and CA films when the extract was incorporated into them, whereas the structure of the biocomposite PPC.CA 90.10 films remained practically unaltered (Figure 2, SEM). The more open structure of PPC and CA containing ORE extract favored the penetration and binding of microorganisms to facilitate the biodegradation process.

In addition, the surface morphology of all bioplastic films after 30 days of BOD experiments was characterized by SEM and shown in Figure 7c. In comparison to the flat and smooth surfaces of the original films (see SI Figure S3), all the bioplastic films after the BOD test showed rougher surfaces with some open cavities as a result of the biodegradation process. Additionally, the chemical structures of bioplastic films before and after 30 days of BOD tests were examined by ^1^H NMR (SI Figure S8). No significant differences were found between the original film and the films after 30 days in seawater, indicating that the biodegradation occurred gradually from the head or the tail of the polymer chains, and the polymer backbone did not change during the first 30 days of biodegradation under seawater. These interesting results reveal good biodegradability of CO_2_-based bioplastic films in a sea environment. Furthermore, the developed PPC.CA bioplastic films are highly biocompatible, as demonstrated from biocompatibility tests using fibroblast cells as models with high cell viability after 3 days of experiments (SI Figure S9).

## 3 Conclusions

We report the development of all natural CO_2_-based flexible and freestanding PPC.CA bioplastic films by an easily scalable and benign solvent casting technique. Thanks to well-distributed CA microparticles within the PPC matrix and their hydrogen bonding interactions, these bioplastics showed improvements in mechanical, thermal, and water vapor barrier properties with respect to the single component polymer films. The great transparency of neat bioplastic films and high UV protection of films containing waste oregano extract are ideal for food packaging application. Their transparency, mechanical properties, and water vapor barrier are comparable to those of commercial nonbiodegradable plastic. In addition to that, the developed PPC.CA biocomposites containing oregano waste extract are biodegradable, biocompatible, and exhibit great antioxidant activity, comparable to popular antioxidant-rich foods, making them suitable candidates for sustainable active food packaging materials.

## 4 Experimental Section

### 4.1 Materials

Oregano byproduct was kindly provided by Kütaş Tarım Ürünleri Dış Tic. Ve San A.Ş., Turkey. Poly(propylene carbonate) (PPC) was purchased from Empower Materials, U.S.A. Cellulose acetate (CA), 2,2-diphenyl-1-picrylhydrazyl free radical (DPPH^•^), Dulbecco’s modified Eagle’s medium (DMEM), Fetal Bovine Serum (FBS), l-Glutamine (200 mM), Trypsin 0.25% (containing 1 mM EDTA), Water-Soluble Tetrazolium Salt (WST-1) assay and Dulbecco’s Phosphate Buffered Saline (DPBS), acetone and ethanol were obtained from Sigma-Aldrich, Italy. All chemicals were analytical grade and used as received without any further purification. Deionized water was obtained from Milli-Q Advantage A10 ultrapure water purification system. NIH/3T3 (murine fibroblasts cell line) was received from American Type Culture Collection (ATCC).

### 4.2 Preparation of Oregano Waste Extract Solution

A mixture containing 5 g of dried and milled oregano byproduct and 50 mL of acetone was added in a vial to obtain the 10% (w/v) concentration of oregano waste in acetone. The use of acetone, a nontoxic solvent, for extraction process has been observed to be more efficient than other alcohol and aqueous based solvent systems for extracting active phenolic antioxidants from plants.^5^ The extraction was perform at room temperature (23–25 °C) on a vortex mixer (Heidolph D-91126, Germany) at 500 rpm for 3 days. After that, the mixture was filtered with Sartorius Stedim folded filter grade 389F (particle retention around 8–12 μm) and stored in the dark for the next steps. The extraction yield was determined gravimetrically after the evaporation of acetone as 7.98 ± 0.14% of dried extract/dried oregano waste.

### 4.3 Preparation of CO_2_-Based Bioplastic Films

CO_2_-derived bioplastic films were fabricated by a simple solvent casting process. PPC and CA mixtures with desired weight proportion were dissolved in acetone in standard glass vials to obtain the respective solutions with a concentration of 10% (w/v). The dissolution of PPC and CA mixtures was carried out at room temperature (23–25 °C) with the help of a vortex mixer (Heidolph D-91126, Germany) at 500 rpm for 2 h to obtain homogeneous solutions. The weight percentages of CA in the bioplastic films ranged from 0 to 20 wt %. Consequently, the solutions were cast in a mold and dried under an aspirated hood. After 24 h, the films were removed from the molds. Thus, a series of freestanding plastic films with an average thickness of 80 μm were prepared. All the samples are denoted as PPC.CA *x*.*y*, where, *x*, *y* are the weight percentages of PPC and CA in the bioplastic films, respectively. Table 2 presents all the composition details of PPC.CA bioplastic films.

**Table 2:** Compositions of Developed PPC.CA Bioplastic Films^a^

sample	% wt poly(propylene carbonate) (PPC)	% wt cellulose acetate (CA)
PPC 100	100	0
PPC.CA 90.10	90	10
PPC.CA 80.20	80	20
CA 100	0	100

a The films containing oregano extract are named as PPC.CA x.y ORE, where x and y are the percentage of PPC and CA in the films, respectively.

In the case of bioplastic films containing oregano waste extract, the fabrication process is identical to the above procedure, however, the oregano waste extract solution was used to dissolve PPC and CA instead of pure acetone. The films incorporating oregano waste extract are named as PPC.CA *x*.*y* ORE, where, *x* and *y* are the weight percentages of PPC and CA in the bioplastic films, respectively. The prepared biocomposite films contain 7.98 wt % of oregano extract with respect to the total weight of the PPC and CA polymers.

### 4.4 Characterization of CO_2_-Derived Bioplastic Films

#### 4.4.1 ATR-FTIR Spectroscopy

The chemical composition and potential intermolecular interactions within bioplastic films were characterized by an attenuated total reflectance (ATR) accessory (MIRacle ATR, PIKE Technologies) coupled to a Fourier transform infrared (FTIR) spectrometer (VERTEX 70v, Bruker). All the spectra were recorded between the 4000 to 600 cm^–1^ range with a resolution of 4 cm^–1^ and 128 repetitive scans.

#### 4.4.2 Morphological and Surface Characterization

The specimens for cross-section morphological observation were fractured inside liquid nitrogen. The films were mounted with the cryo-fractured section up on metallic stubs that have 90° vertically cut surfaces.

All the samples (both film surfaces and cross sections) were coated with 15 nm gold, and their morphologies were investigated by Scanning Electron Microscopy (SEM), using a variable pressure JEOL JSM-6490LA microscope in high vacuum mode and an acceleration voltage of 10 kV.

#### 4.4.3 Optical Characterization

The optical properties of all bioplastic films were analyzed by UV–visible spectroscopy. Square shaped specimens (2 × 2 cm^2^) were cut from the freestanding films and placed in a Varian CARY 300 Scan UV–visible spectrophotometer sample holder. The UV transmittance measurements were carried out in triplicate in the wavelength range of 200–800 nm.

#### 4.4.4 Mechanical Characterization

The tensile measurements were conducted on five specimens for each bioplastic film type, according to ASTM D882 Standard Test Methods for Tensile Properties of Thin Plastic Sheeting with an Instron dual column tabletop universal testing System 3365 using 2 kN load cell, at 25 °C and 50 mm/min cross-head speed.^7,52^ Samples were cut with a standard dog-bone press. The dimensions of the tested region were 25 × 4 mm^2^.

The experimental Young’s moduli were compared to prediction values to profoundly investigate the structure and properties of the PPC.CA interface. Two different models (parallel and series) based on the rule of mixtures were employed to predict the mechanical behaviors of PPC.CA composites. In the parallel model, two polymers in the composites connect parallel to each other, and as a result, the applied stress during the mechanical test elongates both polymers to the similar extension. The highest upper bound given by the parallel model of the Young’s modulus prediction is calculated as below:^41−43^
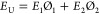
3where, *E*_U_ is the highest upper bound of the Young’s modulus. *E*_1_ and *E*_2_ are the measured Young’s modulus values of PPC and CA films, respectively. Ø_1_ and Ø_2_ are the corresponding volume fraction of PPC and CA in the biocomposite films.

In the series model (Reuss prediction), the polymer components are assumed to be arranged in series perpendicular to the applied force direction. The lowest lower Young’s modulus prediction from this model is identified from following formula:^41−43^
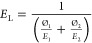
4where *E*_L_ is the lowest lower bound of the Young’s modulus. *E*_1_ and *E*_2_ are the measured Young’s modulus values of PPC and CA films, respectively. Ø_1_ and Ø_2_ are the corresponding volume fractions of PPC and CA in the biocomposite films.

#### 4.4.5 Thermogravimetric Analysis (TGA) Measurement

The thermal stability of PPC.CA films were characterized by means of TGA using a Mettler Toledo TGA/DSC1 STARe System. The measurements were performed on 3–5 mg samples in an aluminum pan at a heating rate of 10 °C/min, from 30 to 600 °C in nitrogen atmosphere.

#### 4.4.6 Water Vapor Permeability

Water vapor permeability (WVP) was determined according to the ASTM E96 standard method.^7^ Bioplastic films were mounted on the top of permeation cells with 7 mm internal diameter and 10 mm inner depth containing 400 μL of deionized water. The permeation cells were placed in 0% relative humidity (RH) desiccator with anhydrous silica gel used as a desiccant agent.^7,28^ The amount of water transferred through the film was determined from the weight change of the permeation cell every hour for 8 h. The weight loss of the permeation cells were plotted as a function of time. The slope of each line was calculated by linear fit. The water vapor transmission rate (WVTR) and WVP of the bioplastics were calculated as follows:^7,28,53^
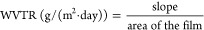
1
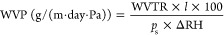
2where, *l* (m) is the film thickness, ΔRH (%) is the percentage relative humidity gradient, and *p*_s_ (Pa) is the saturation water vapor pressure at 25 °C (3168 Pa^7^).

#### 4.4.7 Water Contact Angle Measurement

Wetting characteristics of bioplastic films were determined by water contact angle measurements using a Theta Optical Tensiometer (Dataphysics OCAH200) with 3 μL deionized water droplets. Contact angles were measured with ten repetitions for each sample on different sample surface regions, and the average values are reported. The static water contact angle can be found in SI Figure S6.

#### 4.4.8 Antioxidant Test (DPPH^•^ Free Radical Scavenging Assay)

The antioxidant characteristics of the bioplastic films containing oregano waste extract were determined by the standard DPPH^•^ free radical scavenging method.^3,54−56^ Preweighted PPC.CA films (0.02 g) were placed in separate vials. A 0.1 mM solution of DPPH^**•**^ in ethanol (2.0 mL) was added to the vial, and it was sealed and kept in the dark. A specific preset reaction time was assigned to each vial. When this reaction time was completed, the films were removed and the solution was measured by UV–vis spectroscopy to determine the absorbance (*A*_1_) at 517 nm. The reference (control measurements) absorbance value (*A*_2_) was measured with only 2.0 mL of 0.1 mM DPPH^•^ in ethanol solution. The UV–vis absorbance of the solutions for antioxidant assays was measured by Varian CARY 300 Scan UV–vis spectrophotometer in the wavelength range of 200–800 nm. For comparison purposes, the antioxidant activities of the PPC.CA films without oregano waste extract were also measured and found to show negligible activities with respect to one of films containing oregano waste extract. Percent DPPH^•^ free radical scavenging activity was calculated by the following formula:^3,29,56,57^
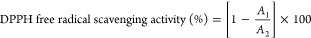
5where, *A*_1_ is the absorbance of the solution containing the PPC.CA films and DPPH^**•**^ radical, and *A*_2_ refers to the absorbance of DPPH^**•**^ control solution, both determined at 517 nm.

#### 4.4.9 Biochemical Oxygen Demand (BOD) Analysis

Underwater biodegradation potential of the CO_2_-based bioplastics was determined using standard BOD tests. 200 mg of samples were finely cut and placed in brown glass bottles along with 432 mL of seawater (which was collected from a local coastal region in Genoa, Italy in May, 2019). The bottles were sealed with OxiTop caps and stirred with magnetic anchors for 30 days in the dark, mimicking the pelagic marine environment.^32,58^ The amount of consumed oxygen in the degradation reaction was recorded every 6 h by the sensor mounted in the OxiTop cap. Three bottles with only seawater were also measured as control. The bioplastic residues after 30 days BOD experiments were characterized by SEM and ^1^H NMR (see SI).

#### 4.4.10 Proton Nuclear Magnetic Resonance (^1^H NMR) Measurement

The ^1^H NMR spectra were measured using Bruker Ultra Shield Avance spectrometers 400 MHz with deuterated acetone as a solvent. The ^1^H NMR spectra are shown in SI Figure S8.

#### 4.4.11 X-ray Diffraction (XRD) Spectroscopy

XRD measurement was carried out to analyze the change in crystallinity of the bioplastic films using a Rigaku SmartLab X-ray diffractometer equipped with a copper rotating anode. The measurements were performed using a 2θ scan. The obtained XRD patterns are presented in SI Figure S4.

#### 4.4.12 Cell Culture

NIH/3T3 fibroblasts were grown in T75 culture flasks in DMEM supplemented with 10% (v/v) of FBS and 1% (v/v) of l-glutamine in a humidified incubator at 37 °C and with 5% CO_2_. Culture medium was replaced every 3 days. When cultured cells were approximately 80% confluent, they were passaged by a 3 min exposure to 0.25% trypsin.

#### 4.4.13 Assessment of Cell Viability: WST-1 Assay

To evaluate the biocompatibility of pure PPC100, CA100 along with PPC.CA 90.10 and 80.20 bioplastic films, an indirect cytotoxicity test was performed according to the ISO10993-5 standard test. To prepare extract media, the bioplastic films were sterilized under UV light for 30 min/side and incubated in cell culture medium for 24 h at 37 °C in a humidified atmosphere (5% CO_2_). The cytotoxicity of the extract medium on cells was evaluated by the WST-1 assay, a colorimetric assay based on oxidation of tetrazolium salts.

Murine fibroblast (NIH/3T3) cells were seeded in 96-well plates at a density of 10.000 cells/well and incubated overnight for cell adhesion. The medium was then replaced with the extraction one and the cells were incubated for a further 1 or 3 days. After the treatment, cells were rapidly rinsed with prewarmed PBS, and the extraction medium was replaced with a fresh one, followed by the addition WST-1 reagent, diluted 1:10 into each well. Cells were further incubated for 3 h and then absorbance was read by a microplate reader (Supplier) at 450 nm.

Cell viability was expressed as percentage survival relative to control cells and an extract medium without cells was used as a control for the absorbance reading. All the results were normalized against the absorbance of blank wells. The biocompatibility results of PPC.CA bioplastic films can be found in SI Figure S9.

#### 4.4.14 Statistical Analysis

All the measured values were expressed as mean  ±  standard error of the mean. For in vitro biocompatibility tests One-way ANOVA was used to evaluate the statistical significance, followed by Bonferroni’s post hoc test using GraphPad Prism 5 (GraphPad Software Inc. San Diego, CA, U.S.A.). A *p* value of less than 0.05 was considered to be statistically significant.

## Supporting Information Available

FTIR spectra of biocomposite films without oregano waste extract; FTIR spectra of biocomposite films containing oregano waste extract; surface morphologies, XRD analysis, tensile strain–stress curves, and static water contact angle of biocomposite films; calibration curves of DPPH^•^ scavenging activities; ^1^H NMR spectra films before and after 30 days of biodegradation tests; and viability of NIH/3T3 fibroblasts after 1 and 3 days of exposure to the biocomposites extraction media (PDF)
