# Supplementary material for: Transparent Bioplastic Derived from CO2-Based Polymer Functionalized with Oregano Waste Extract toward Active Food Packaging
Source: ACS Appl Mater Interfaces. 2020 Sep 21;12(41):46667–77. doi: 10.1021/acsami.0c12789 (PMC8011794; doi:10.1021/acsami.0c12789)
Supplement: Supplementary file 1 — am0c12789_si_001.pdf [file am0c12789_si_001.pdf]

Transparent Bioplastic Derived from CO_2-Based Polymer Functionalized with Oregano Waste Extract toward Active Food Packaging

Thi Nga Tran,[a,*] Binh T. Mai,[b] Chiara Setti,[a] and Athanassia Athanassiou[a,*]

[a] Smart Materials, Istituto Italiano di Tecnologia, Via Morego, 30, Genova 16163, Italy

[b] Istituto Italiano di Tecnologia, Via Morego, 30, Genova 16163, Italy

Emails: thinga.tran@iit.it; athanassia.athanassiou@iit.it

Supporting Information

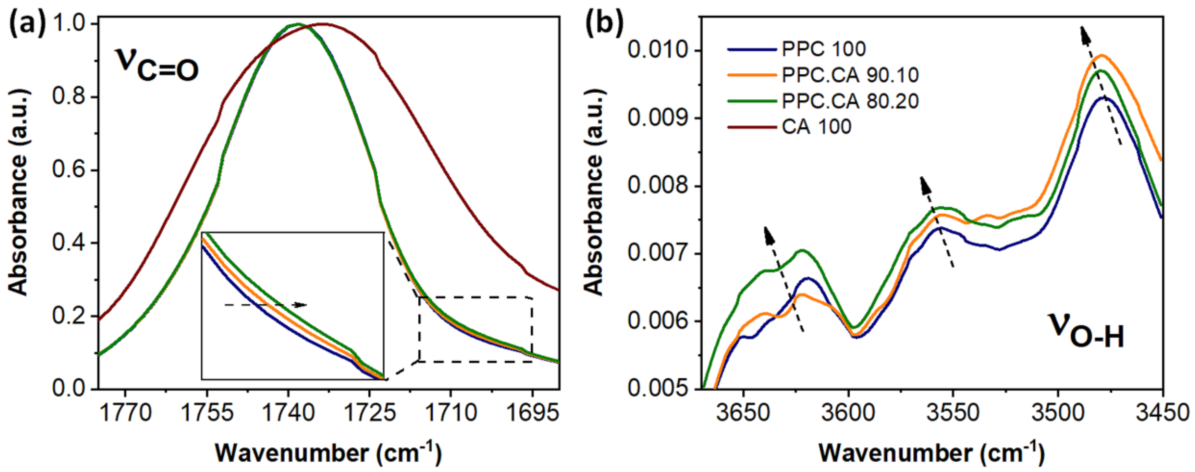

Figure S1. FTIR spectra of biocomposite films without oregano waste extract in: (a) the carbonyl stretching region and (b) the hydroxyl stretching region. The black arrows in the in insets of (a-b) indicate the shifting of these peaks.

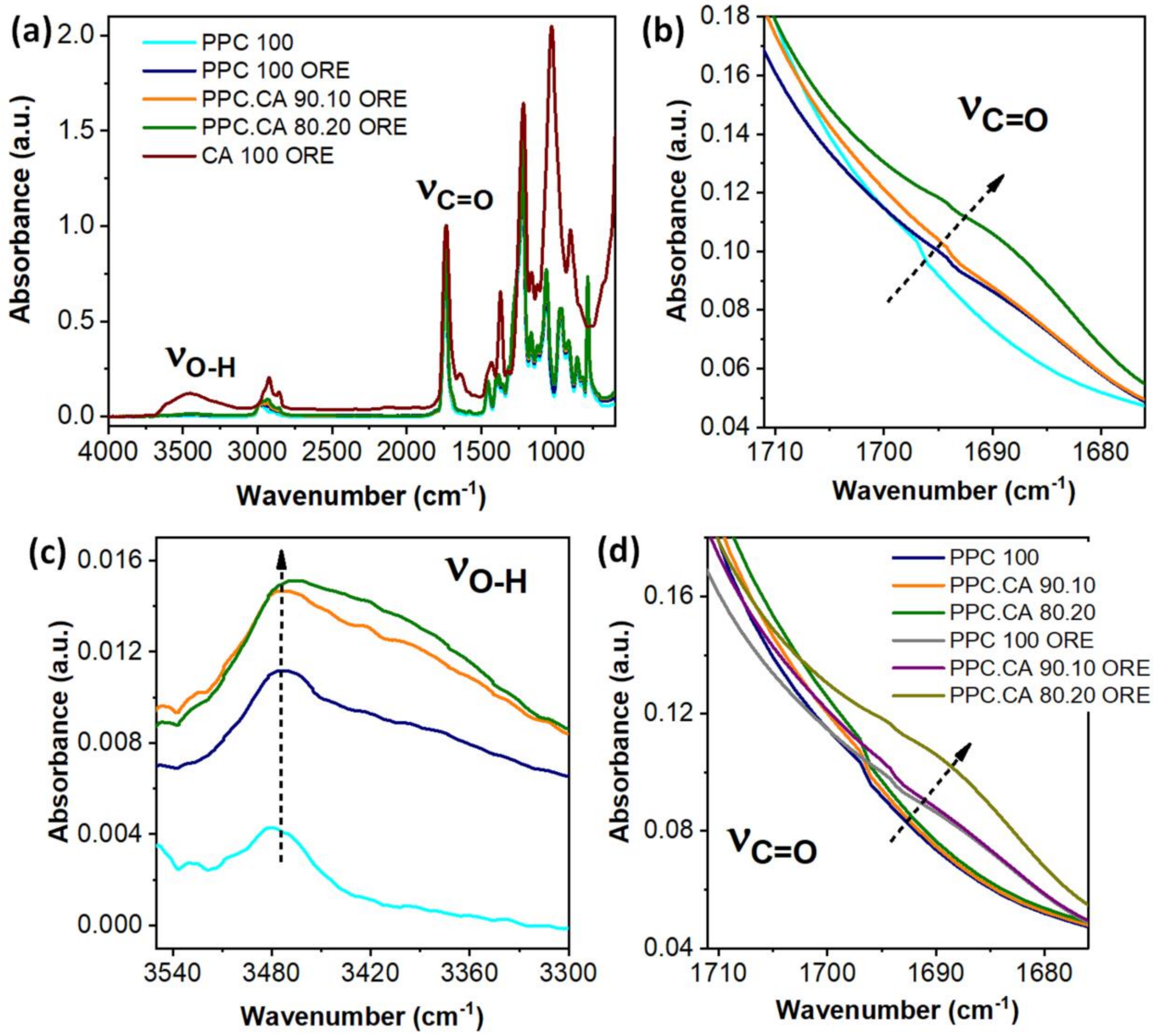

Figure S2. (a) Full FTIR spectra of pure PPC film and PPC.CA biocomposite films containing oregano waste extract. The corresponding FTIR spectra in: (b) the carbonyl stretching region and (c) the hydroxyl stretching region. (d) The detailed FTIR spectra in the carbonyl stretching region of PPC and PPC.CA biocomposite films with and without oregano waste extract. The black arrows in the in insets of (b-d) indicate the shifting of these peaks.

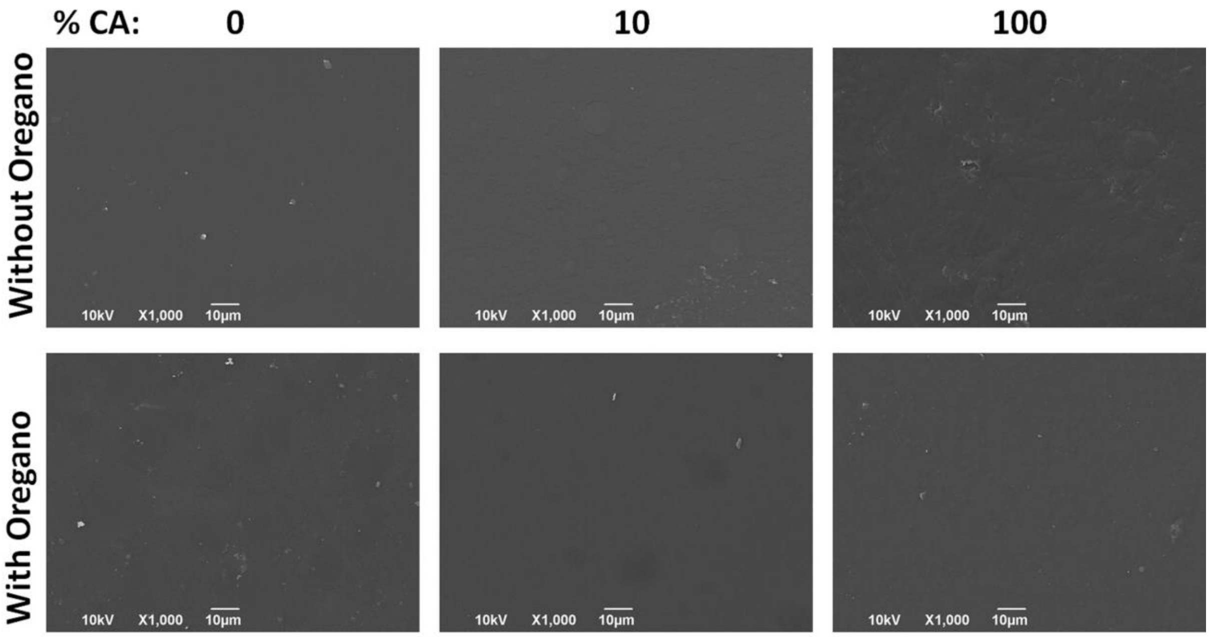

Figure S3. Surface morphologies of pure PPC, pure CA and PPC.CA bioplastic films as well as films containing oregano extract.

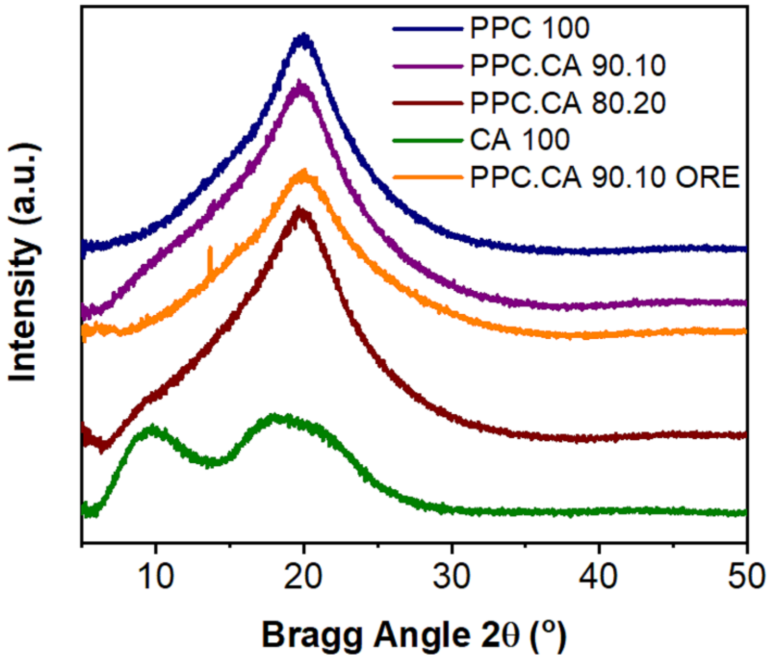

Figure S4. XRD analysis of pure PPC, pure CA and different PPC.CA biocomposite films.

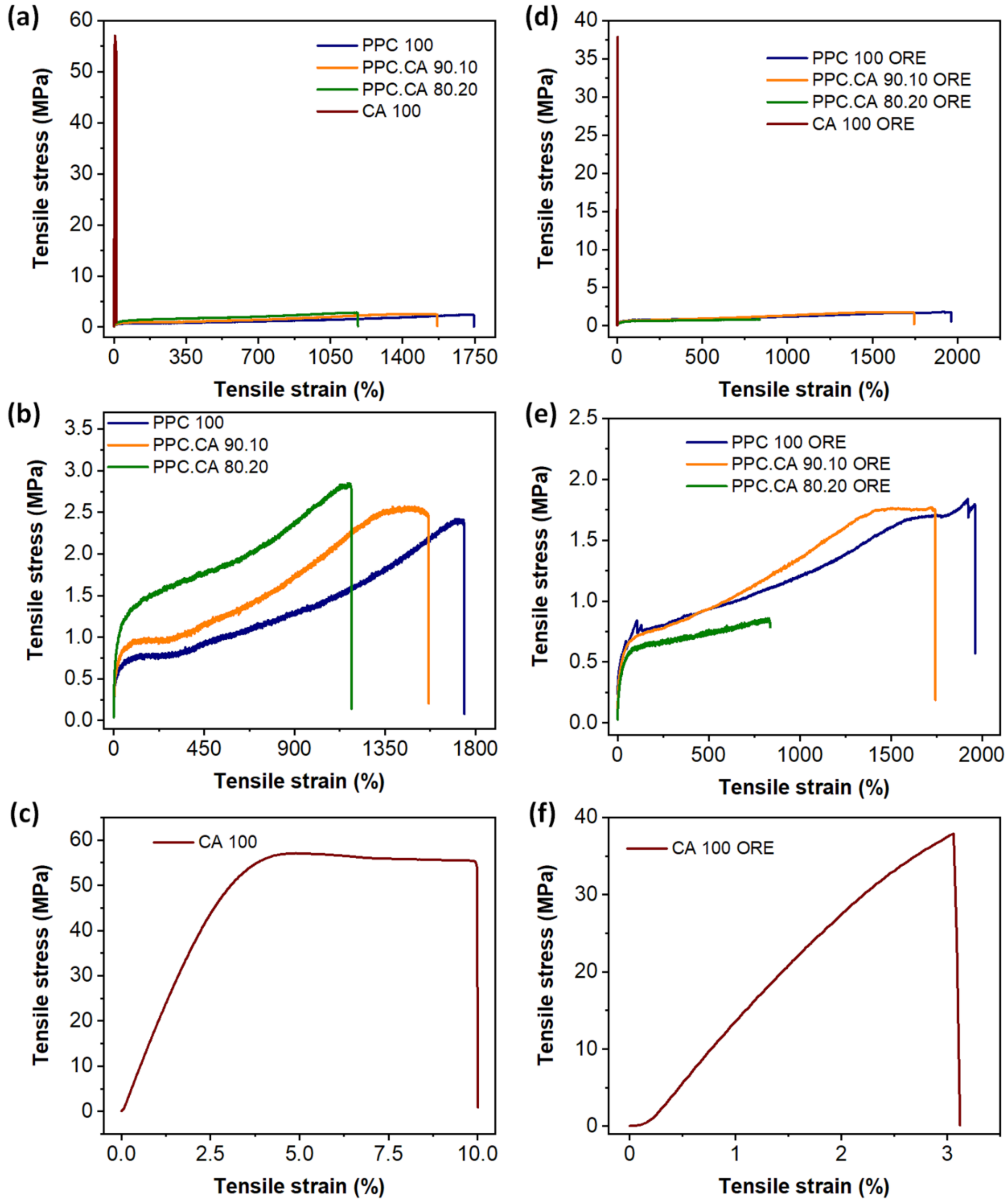

Figure S5. Tensile strain-stress curves of pure PPC, pure CA and PPC.CA bioplastic films as well as films containing oregano extract.

Water contact angle

The behavior of CO_2-based bioplastic films when in contact with water droplets was investigated by measuring the static water contact angle. The average water contact angles

(WCA) of pure PPC, pure CA and PPC.CA films are presented in Fig. S4. The insets in Fig. S4 show the images of water droplet on the surfaces of bioplastic films. The WCA of pure PPC and CA films are 89.4±4.3° and 70.2±5.4°, respectively, indicating quite hydrophobic and quite hydrophilic nature of PPC and CA, respectively. In comparison to neat films, the PPC.CA films have higher WCA, in particular 102.9±4.4° and 101.4±3.0° for PPC.CA 90.10 and 80.20, respectively. These results could be explained by the formation of microparticles of CA inside PPC matrix (Fig. 2 a-h, SEM) leading to moderately rougher film surface, consequently, leading to increased WCA, according to the Wenzel rule for hydrophobic surfaces.[1] When the oregano extract was introduced, the WCA of PPC100 ORE and CA100 ORE were increased to 99.5±2.0° and 85.0±9.1°, correspondingly. The PPC.CA 90.10 ORE and 80.20 ORE show WCA at 86.3±5.6° and 84.6±6.2°, which are reduced compared to the corresponding films without oregano extract. Overall, all PPC.CA bioplastic films with oregano extract show WCAs around 90°, indicating their sufficient hydrophobicity to be used in food packaging application.

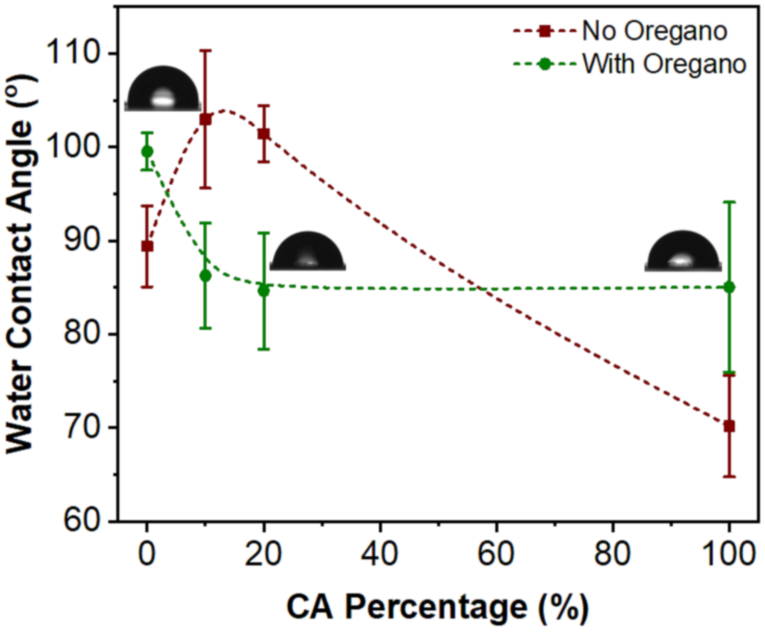

Figure S6. Static water contact angle of pure PPC, pure CA and different PPC.CA biocomposite films and the films with oregano waste extract. The insets are the photos of water droplets on corresponding samples.

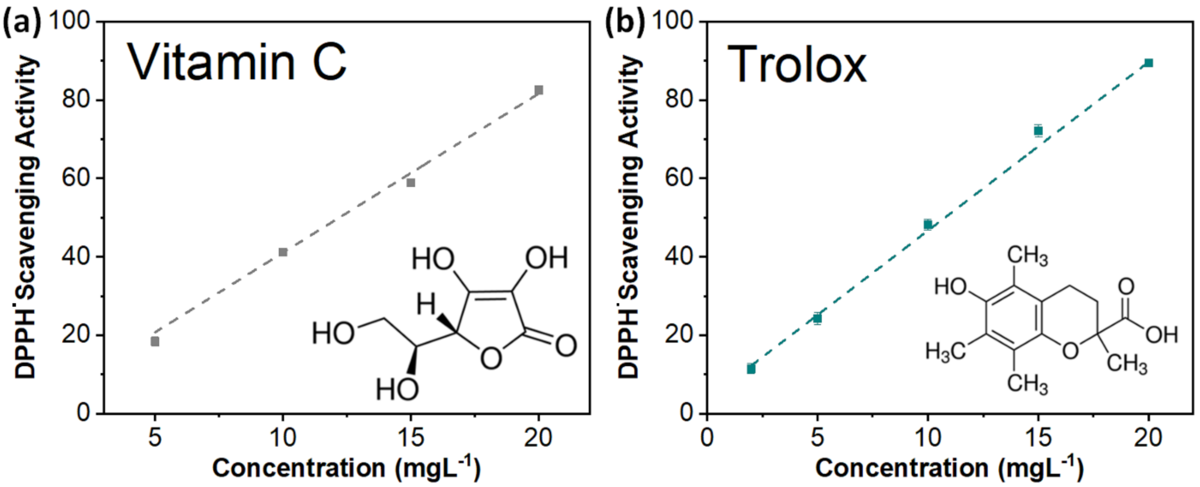

Figure S7. Calibration curves of DPPH$^{\bullet}$ scavenging activities of (a) Vitamin C and (b) Trolox at various concentrations.

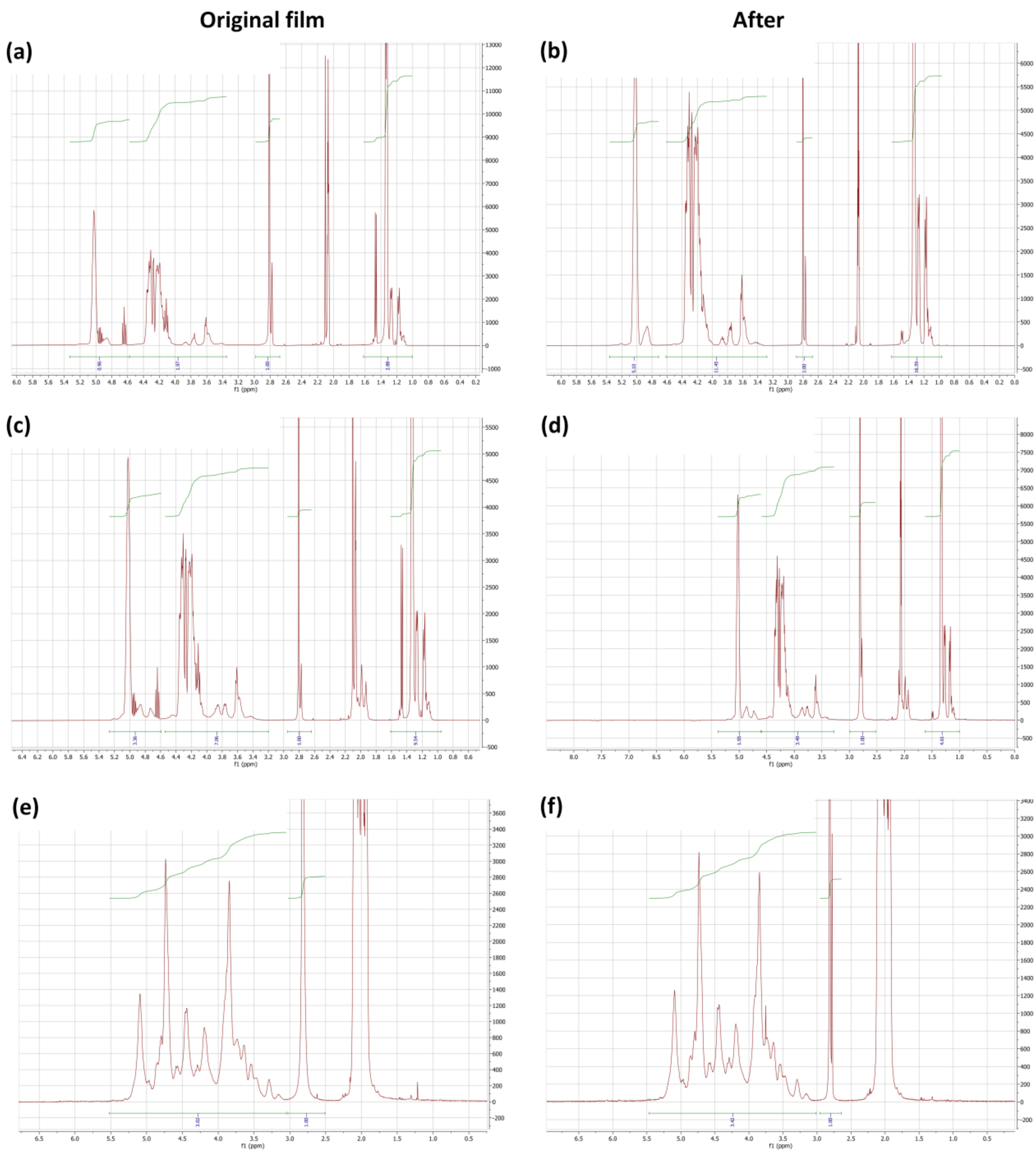

Figure S8. ^{1}H NMR spectra of pure PPC, pure CA and PPC.CA 90.10 before and after 30 days of biodegradation tests.

Biocompatibility tests

The biocompatibility of pure PPC100, CA100 polymeric films and their blends (PPC.CA 90.10 and 80.20) was assessed using fibroblast cells as model. Figure S7 displays WST-1 assay outcomes after 1 and 3 days of cell culture, revealing that the PPC.CA bioplastic films were

highly biocompatible, with no statistically significant differences (P > 0.05) in the fibroblast viability for the treated samples in comparison with the controls. These results prove that the PPC.CA films are biocompatible and could be employed for food packaging applications.

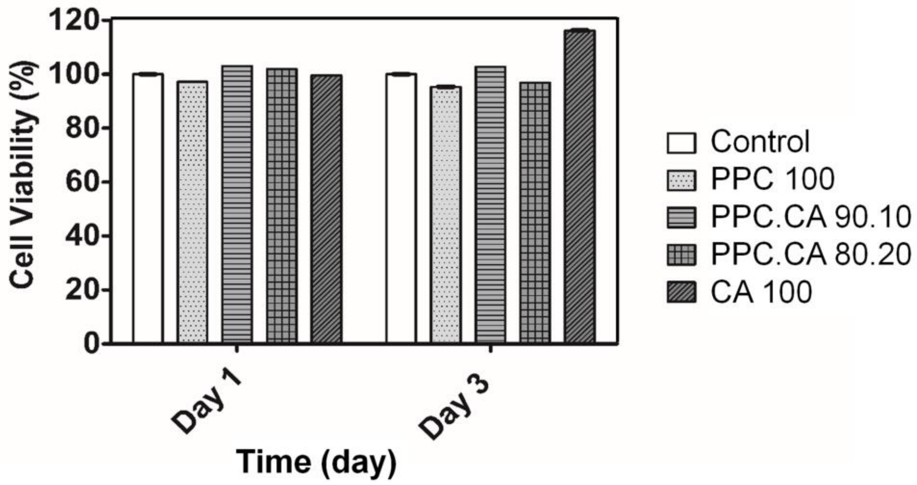

Figure S9. Viability of NIH/3T3 fibroblasts after 1 and 3 days of exposure to the samples extraction media. Survival values are normalized in respect to the control (set to 100%). Average percentage values ± standard deviation of three independent experiments, each performed in three technical replicates, are shown.

References

(1) Yeh, K.-Y.; Chen, L.-J.; Chang, J.-Y. Contact Angle Hysteresis on Regular Pillar-Like Hydrophobic Surfaces Langmuir 2008, 24 (1), 245-251.
